# CpG-ODN Induces a Dose-Dependent Enrichment of Immunological Niches in the Spleen and Lungs of Neonatal Chicks That Correlates with the Protective Immunity against *Escherichia coli*

**DOI:** 10.1155/2020/2704728

**Published:** 2020-01-13

**Authors:** Thushari Gunawardana, Khawaja Ashfaque Ahmed, Kalhari Goonewardene, Shelly Popowich, Shanika Kurukulasuriya, Ruwani Karunarathana, Lisanework E. Ayalew, Ashish Gupta, Betty Lockerbie, Marianna Foldvari, Suresh K. Tikoo, Philip Willson, Susantha Gomis

**Affiliations:** ^1^Department of Veterinary Pathology, Western College of Veterinary Medicine, University of Saskatchewan, Saskatoon, SK, Canada S7N 5B4; ^2^School of Pharmacy, University of Waterloo, 200 University Avenue West, Waterloo, ON, Canada N2L 3G1; ^3^Vaccinology and Immunotherapy, School of Public Health, University of Saskatchewan, Saskatoon, SK, Canada S7N 5E3; ^4^Canadian Centre for Health and Safety in Agriculture, University of Saskatchewan, Saskatoon, SK, Canada S7N 5E5

## Abstract

Immunoprotective function of oligodeoxynucleotides containing CpG motifs (CpG-ODN) has been demonstrated in neonatal chickens against common bacterial pathogens such as *E.coli* and *Salmonella* sp. Our recent study reported that CpG-ODN administration enriches immune compartments in neonatal chicks. However, a causal relationship between CpG-ODN-induced immune enrichment and protective mechanisms remains unestablished. In this study, we investigated *in ovo* administered CpG-ODN-mediated immune cell recruitment in the immunological niches in lymphoid (spleen) and nonlymphoid (lungs) organs using various doses of CpG-ODN and examined whether the immunological profiles have any correlation with immunoprotection against *E.coli* infection. Eighteen-day-old embryonated eggs were injected with either 5, 10, 25, and 50 *μ*g of CpG-ODN or saline (*n* = ~40 per group). On the day of hatch (72 hr after CpG-ODN treatment), we collected the spleen and lungs (*n* = 3‐4 per group) and examined the recruitment of macrophages/monocytes, their expression of MHCII and CD40, and the number of CD4^+^ and CD8^+^ T-cell subsets in the immunological niches in the spleen and lungs using flow cytometry. We observed the dose-dependent recruitment of immune cells, wherein 25 *μ*g and 50 *μ*g of CpG-ODN induced significant enrichment of immunological niches in both the spleen and the lungs. Four days after the CpG-ODN treatment (1-day after hatch), chicks were challenged with a virulent strain of *E. coli* (1 × 10^4^ or 1 × 10^5^ cfu, subcutaneously). Clinical outcome and mortality were monitored for 8 days postchallenge. We found that both 25 *μ*g and 50 *μ*g of CpG-ODN provided significant protection and reduced clinical scores compared to saline controls against *E. coli* infection. Overall, the present study revealed that CpG-ODNs orchestrate immunological niches in neonatal chickens in a dose-dependent manner that resulted in differential protection against *E. coli* infection, thus supporting a cause and effect relationship between CpG-ODN-induced immune enrichment and the antibacterial immunity.

## 1. Introduction

Infectious diseases of neonatal poultry are common due to the immaturity of the immune system or inadequate sensitization of the immune system to antigens [1]. During the first week of a bird's life, high mortality associated with bacterial infections, *Escherichia coli* septicemia in particular [2], has devastating impacts on poultry production [3]. Antimicrobials are effective in controlling bacterial diseases, and thus, the prophylactic use of antimicrobials is a common practice in the poultry industry [4]. However, these prophylactic use of antibiotics in the poultry industry may lead to antibiotic residues in poultry products [5, 6] and the emergence of antibiotic-resistant strains of bacteria [4, 7]. Hence, reduction of antimicrobial use is a priority of the poultry industry. Since the use of category1 antibiotics has been discontinued since 2014 [8], the poultry industry needs suitable alternatives to antibiotics for controlling diseases in neonatal chickens [9, 10].

Innate immunity is the first line of defense against infectious agents. The host needs to identify an invading pathogen to mount a rapid immune response. The cells of the innate immune system rely on a set of pattern recognition receptors (PRR), which can detect specific molecular structures present in the pathogens known as pathogen-associated molecular patterns (PAMPs) [11]. The innate immune cells, like dendritic cells (DC), are activated following the interaction of PRR of DC with PAMP of a pathogen. Such PRR-PAMP interaction initiates cell signaling that mounts immune responses, eventually leading to the development of adaptive immunity against the invading pathogen. Toll-like receptors (TLRs) are the main PRR, which are important in the induction of innate immunity [12]. The components of pathogens such as lipopeptides, glycerophosphatidylinositol, lipopolysaccharides (LPS), microbial nucleic acids (dsRNA, ssRNA, and unmethylated CpG DNA), and microbial proteins (flagellin, profilin) are some of the well-known TLR ligands (PAMPs). Many potential TLR agonists have been suggested as immune modulators by different studies [13]. Synthetic CpG-ODNs are recognized by TLR-9 and TLR-21 in mammals and avian, respectively [14–18]. CpG-ODNs initiate immune responses in mice [19], fish [20], cattle and sheep [21], human [22–24], and chickens [25, 26]. CpG-ODNs are safe immunoprotective agents, and the Food and Drug Administration has approved its use in humans [27].

In quest of an alternative to antimicrobial agents against bacterial infections, our laboratory pioneered the use of CpG-ODN alone as an immune protective agent against *E. coli* infection in chickens [28, 29]. We demonstrated that CpG-ODN administered through various routes protects chickens against *E. coli* [4, 26] and *Salmonella* Typhimurium infection [29, 30]. The protective effect of CpG-ODN has also reported against *Salmonella* Enteritidis infection by other studies [31, 32]. Moreover, CpG-ODN formulation with nanoparticles further improved its immunoprotective action [30, 33, 34]. Several studies have also reported improved immune responses of nanoparticle formulated CpG-ODN [17, 18] and demonstrated enhanced expression of cytokines and chemokines following CpG-ODN administration in chickens [34, 35]. The previous study showed that the resolution of *Salmonella* Typhimurium infection strongly correlated with proinflammatory cytokine expression in chickens [36]. Despite recent advances, the immunoprotective mechanism(s) of CpG-ODN alone against bacterial infections remained poorly understood.

Proinflammatory cytokines stimulate the secretion of chemokines and expression of cell-surface leukocyte adhesion molecules and promote the rapid recruitment of immune cells in the inflammatory area [37–39]. We found that the intrapulmonary delivery of CpG-ODN initiated the infiltration of inflammatory cells in the pulmonary parenchyma [4]. We recently found that *in ovo* administration of CpG-ODN enriches various immune compartments in neonatal chicks [40]. We hypothesize that CpG-ODN-mediated protection may be through the regulation of immunological niches in neonatal chickens. Thus, the objective of this study was to evaluate the influx of macrophages and CD4^+^ and CD8^+^ T-cell subsets in the immunological niches such as the spleen and lung in chickens and investigate if immune profiles correlate with the CpG-ODN-induced protection against *E. coli* infection.

## 2. Materials and Methods

### 2.1. Experimental Chickens

All animal experiments were approved by the University of Saskatchewan's Animal Research Ethics Board and adhered to the Canadian Council on Animal Care guidelines for humane animal use. Fertilized hatching eggs were obtained from a commercial broiler breeder operation in Saskatchewan, Canada. Eggs were incubated at the Animal Care Unit (ACU) at the Western College of Veterinary Medicine, University of Saskatchewan. Following *in ovo* injections, eggs were transferred to the hatcher in separate groups. No significant differences were seen in fertility and hatchability due to *in ovo* injections between groups. Groups of hatched chicks were tagged for group identification and allocated into an animal isolation rooms at the ACU. Water and commercial broiler rations with no antibiotics were provided *ad libitum* to all groups in the same manner. Each room was ventilated with filtered, nonrecirculated air at a rate of 10–12 changes/hr. Air pressure differentials and strict sanitation were maintained in this isolation facility.

### 2.2. Synthetic CpG-ODN

The sequence of CpG-ODN (class B CpG 2007) used was 5′-TCGTCGTTGTCGTTTTGTCGTT-3′. ODNs were produced with a phosphorothioate backbone (Operon Biotechnologies, Inc. Huntsville, AL).

### 2.3. Delivery of CpG-ODN by In Ovo Route

Embryonated eggs (incubated for 18 days) received either 50, 25, 10, or 5 *μ*g of CpG-ODN diluted in sterile pyrogen-free saline, in a total of 100 *μ*l/egg or 100 *μ*l of sterile saline (*n* = ~40/group). Injections were administered by the *in ovo* route into the amniotic cavity through the air cell side of the egg using a 22-gauge, 1-inch hypodermic needle. The volume of the injection and the length of the needle were selected to simulate the standard *in ovo* injection technology used in the poultry industry. Following *in ovo* injection, the injection sites of eggs were covered with melted paraffin applied with a wooden applicator and transferred to the hatcher until hatch.

### 2.4. Tissue Sample Collection and Flow Cytometry

Three or four embryos from each group were humanely euthanized at 72 hrs post *in ovo* injections by cervical dislocation and necropsied for tissue collection. Spleen and lung tissues were collected into 1.5 ml microcentrifuge tubes. Cell preparation and antibody staining for flow cytometry were done as previously described with some modifications [41, 42]. Briefly, each spleen was gently pushed through a metal strainer by manual pressure to obtain a single cell suspension with ~10 ml of phosphate-buffered saline (PBS) and collected to a 15 ml centrifuge tube. For lung, each tissue was manually dissected and incubated with ~1 ml of collagenase type 4 (Sigma-Aldrich, St. Louis, Missouri, USA) (1 mg/ml) dissolved in Dulbecco's modified Eagle medium (DMEM) for 30 minutes in 37°C; after incubation, these tissues were filtered through a metal strainer to obtain a single cell suspension and washed twice with PBS. Spleen and lung cells were then incubated with RBC lysis buffer to lyse red blood cells. Following three washes with wash buffer (PBS containing 2% fetal bovine serum and 0.1% sodium azide), cells were stained with appropriate antibodies.

The cell populations of the spleen and lungs collected at 72 hrs post *in ovo* injections from each group (*n* = 3‐4) were stained for the presence of antigen-presenting cells (APCs), CD4^+^ and CD8^+^ T-cell subsets. For detecting antigen-presenting cells (APCs) and their expression of cellular markers, such as CD40 and MHCII, spleen and lung cells (~5 × 10^5^ cells) were incubated with mouse anti-chicken CD40 primary antibody (clone AV79; Bio-Rad, Raleigh, NC, USA) at 4°C for 30 min followed by three washing steps before incubating with goat anti-mouse IgG-PerCP/Cy5.5 secondary antibody at 4°C for 30 min. After three washes, the cells were stained with mouse anti-chicken monocyte/macrophage-PE (clone: KUL01; Southern Biotechnology, Birmingham, Ala, USA) and mouse anti-chicken MHCII-AF488 (clone: 2G11; Southern Biotechnology, Birmingham, Ala, USA) antibody together at 4°C for 30 min. Subsequently, the cells were washed three times and suspended in ~300 *μ*l flow cytometric buffer for the analysis. Another set of spleen and lung cells (~5 × 10^5^ cells) were incubated with anti-chicken CD8-FITC (clone: EP72; Southern Biotechnology, Birmingham, Ala, USA) and CD4-PE (clone: CT-4; Southern Biotechnology, Birmingham, Ala, USA) together at 4°C for 30 min to determine CD4^+^ and CD8^+^ T-cells. Following three washes, these cells were also suspended in ~300 *μ*l flow cytometric buffer (PBS containing 2% fetal bovine serum and 0.1% sodium azide) in flow cytometry tubes and processed for flow cytometric analysis. In flow cytometry, cells were gated based on forward vs. side scatter plot. Flow cytometry data were acquired by Epics XL (Beckman Coulter) and FACSCalibur (BD Bioscience), and data were analyzed with FlowJo software (Tree Star).

### 2.5. Bacteria

For the challenge, a field isolate of *E. coli* from a turkey with septicemia was used as previously described [26, 28], which was stored at -80°C in 50% brain-heart infusion broth (BHI; Difco, Detroit, MI) supplemented with 25% (*w*/*v*) glycerol (VWR Scientific, Inc., Montreal, Quebec). This *E. coli* strain was serogroup O2, nonhemolytic, with a K1 capsule and Type 1 pili, produced aerobactin and serum resistant. First, bacteria were cultured on Columbia sheep blood agar plates for 18-24 hrs at 37°C. Then, one colony was added to 100 ml of Luria broth in a 250 ml Erlenmeyer flask. The culture was grown at 37°C for 16-18 hrs with shaking at 150 rpm, and optical density (OD) was measured at 600 nm wavelength using a spectrophotometer. This stationary phase culture contained approximately 1 × 10^9^ colony-forming units (cfu) of bacteria per ml. Finally, cultures were diluted in sterile saline so the concentration of bacteria required for the challenge (1 × 10^5^ or 1 × 10^4^ cfu/in 250 *μ*l bird) was obtained. Viable bacterial counts were determined by counting the number of colonies following plating serial dilutions of the challenge doses in duplicate on Columbia sheep blood agar plates and incubating for 18-24 hrs at 37°C.

### 2.6. *E. coli* Challenge Experiment

All the remaining birds (bird numbers per group; CpG-ODN 5 *μ*g: *n* = 37; CpG-ODN 10 *μ*g: *n* = 33; CpG-ODN 25 *μ*g: *n* = 34; CpG-ODN 50 *μ*g: *n* = 30, saline: *n* = 35) received either 1 × 10^5^ (50% of birds in each group) or 1 × 10^4^ (50% of birds in each group) cfu of stationary phase *E. coli*, in a total volume of 250 *μ*l per bird, by subcutaneous injection in the neck one day posthatch. *E. coli* septicemia with airsacculitis, pericarditis, or perihepatitis develops in 60%–90% of birds that are not protected by treatment intervention, in this model. Clinical signs, pathology, bacterial isolations from the air sacs, and mortality were observed and recorded every day for 8 days following challenge with *E. coli*. Birds were evaluated three times daily at the critical stage, which is the first four days postchallenge, then twice daily for 7 days postchallenge. Birds were observed for clinical signs, and each individual was assigned a daily clinical score: 0 = normal; 0.5 = slightly abnormal appearance, slow to move; 1 = depressed, reluctant to move; 1.5 = reluctant to move, may take a drink and peck some; 2 = unable to stand or reach food or water; and 3 = found dead. Birds that received a clinical score of 2 were humanely euthanatized by cervical dislocation. Chicks that were found dead or euthanatized were necropsied, and bacterial swabs were taken from air sacs immediately. On day 8, after the *E. coli* challenge, all remaining birds were euthanatized by cervical dislocation. Bacterial swabs were taken from the air sacs and cultured on Columbia sheep blood agar using a typical method of streaking on four quadrants of the plate of the medium. A semiquantitative estimate of *E. coli* isolation was conducted on Columbia sheep blood agar. Growth on these plates were recorded on a scale from 0 to 4+, where 0 = no growth; 1+ = growth of bacteria on the area 1; 2+ = growth of the bacteria on areas 1 and 2; 3+ = growth of bacteria on areas 1, 2, and 3; and 4+ = growth of bacteria on areas 1, 2, 3, and 4. All lesions found in dead or euthanized chicks were recorded.

### 2.7. Statistical Analysis

Prism 5.0, GraphPad Software Inc., San Diego, CA, was used to analyze and graph survival trends, cumulative clinical scores (CCSs), bacterial percentages, and cell populations from flow cytometry analysis, with a significance level of *P* < 0.05. The survival patterns and median survival times were compared using the log-rank test and the chi-square statistic. The reduction of the relative risk of mortality of groups of birds was calculated using Microsoft excel. The clinical score for each bird in the respective group was summed every day for 8-day observation period to calculate the CCS, and the significance of differences among groups was tested with the use of Kruskal-Wallis nonparametric analysis of variance. For testing significant differences in the means of immune cell numbers and their maturation marker expression between groups, ANOVA testing was done. Dunnett's test was used as a *post hoc* test following ANOVA to assess for significant differences between each treatment group compared to the saline control group. For analyzing the difference in CD4^+^ and CD8^+^ T-cells between groups, we used two-way ANOVA followed with the Bonferroni posttest and the Student *T*-test with Welch's correction for unequal variance was used, with a significant difference of *P* < 0.05.

## 3. Results

### 3.1. Flow Cytometry

Flow cytometry analysis of the lungs and at 72 hrs post *in ovo* injections (on the day of hatch) showed a significant influence of CpG-ODN on immune cell components of birds. Results demonstrated a dose-dependent influence of CpG-ODN affecting cell population percentages and maturation marker expression levels.

### 3.2. Immune Cell Profiling of the Lung

#### 3.2.1. Antigen-Presenting Cells in the Lung

**Figure 1:**
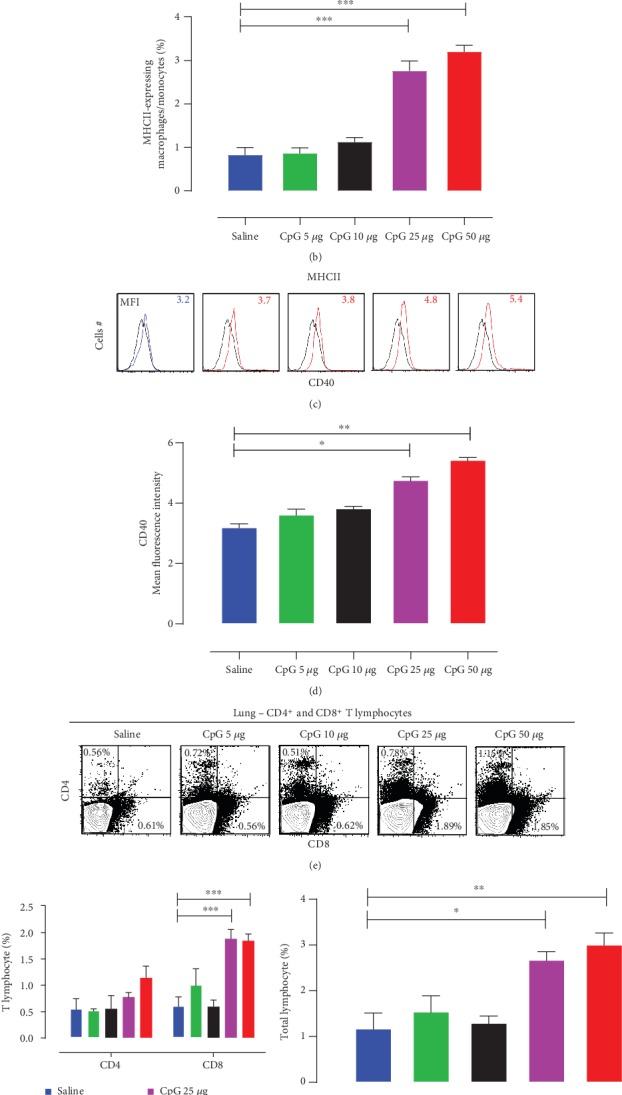
CpG-ODN-induced a dose-dependent enrichment of lung immunological niches. CpG-ODN treatment resulted in an increase in the number of infiltrating antigen-presenting cells and T lymphocytes in the lung. Flow cytometric analysis of lung cells at 72 hrs post *in ovo* injections (day 21 embryo) (*n* = 3‐4/group). First, cells were plotted based on forward vs. side scatter to gate monocyte/macrophage populations. Then, using PE-labeled mouse anti-chicken monocyte/macrophage (clone: KUL01) and AF488-labeled mouse MHC II monoclonal antibodies, the double-positive population was gated to quantify the number of APCs (dot plot panels, a). Bar diagrams (b) show the means of the total percentage of MHC II^+^ APC in various groups. Histogram panels (c) indicate the level of CD40 expression on MHC II^+^ APCs (the MFI is indicated). Black histogram = isotype control, blue = saline, and red = CpG-ODN treated. Bar diagrams (d) show the means of MFI of CD40 expression on MHC II^+^ APC (vertical line and horizontal bar show the standard error of mean-SEM), *n* = 3‐4. Dunnett's test following ANOVA testing was used to test for significant differences between CpG-ODN doses and the saline control group. Asterisks indicate groups that were significantly different from the control group (*P* < 0.05). (MFI—geometric mean fluorescence intensity). (e–g) Flow cytometric analysis of lung T-cell populations in each group (*n* = 3‐4) at 72 hr post *in ovo* injections (day 1 hatch). CD4^+^ and CD8^+^ T-cells in the lung (dot plot panels, e) were quantified using PE-labeled mouse anti-chicken CD4 and FITC-labeled mouse anti-chicken CD8 monoclonal antibodies. Bar diagrams show CD4^+^ and CD8^+^ T-cell number in the lungs (f), and the total number of CD4^+^ T-cells and CD8^+^ T-cells combined (g) in various groups. Two-way ANOVA following the Bonferroni posttest was done when CD4^+^ and CD8^+^ cells were compared between CpG-ODN received groups and saline control. Dunnett's test following ANOVA testing was used to test for significant differences in total T-cells between different CpG-ODN doses and the saline control group. Vertical line and horizontal bar show the standard error of mean-SEM. Asterisks indicate groups that were significantly different from the control group, ^∗^*P* < 0.05, ^∗∗^*P* < 0.01, and ^∗∗∗^*P* < 0.001.

CpG-ODN showed a strong effect on MHCII-expressing APC cell population (monocyte/macrophages) 72 hrs post *in ovo* injections. The groups that received 25 *μ*g or 50 *μ*g of CpG-ODN per bird showed a significant increase in the lung APC population compared to the saline control group (~3 and ~4 times higher, respectively) (Figures 1(a) and 1(b)). The highest APC percentage was seen with 50 *μ*g of CpG-ODN and lowest with the saline control. However, 5 *μ*g and 10 *μ*g of CpG-ODN treatment did not show a significant effect on APC population compared to the saline-treated control group in the lungs (Figures 1(a) and 1(b)). APCs (monocyte/macrophages) were further analyzed for the expression of maturation markers (CD40 costimulatory molecule) (Figure 1(c)). The CD40 signaling is well known to activate APCs and facilitate T-cell priming [43] to generate protective CD8^+^ cytotoxic T-cell (CTL) immunity. The mean fluorescence intensity (MFI) of CD40 expression on APCs, with each CpG-ODN dose tested, is shown in histograms (Figure 1(c)) and in the graph (Figure 1(d)). The MFI increased in a dose-dependent manner and was significantly high in both 25 *μ*g and 50 *μ*g groups (Figure 1(d)).

#### 3.2.2. CD4^+^ and CD8^+^ T-Cell Subsets in the Lung

CD4^+^ (T helper cells) and CD8^+^ (cytotoxic T-cells) cells are the main types of T lymphocytes which play an important role in both humoral and cell-mediated immunity. We evaluated the effect of CpG-ODN on CD4^+^ and CD8^+^ cell populations in the chicken lungs 72 hrs post-CpG-ODN in ovo injections. Both CD4^+^ and CD8^+^ cell populations increased after CpG-ODN administration in a dose-dependent manner in the lungs (Figures 1(e)–1(g)). A significant increase in CD8^+^ T-cells (Figure 1(f)), as well as a total number of T lymphocytes (CD4^+^ and CD8^+^ T-cells combined) (Figure 1(g)), was found in the lungs of chickens that received 25 *μ*g or 50 *μ*g CpG-ODN.

#### 3.2.3. Histological Evaluation

**Figure 2:**
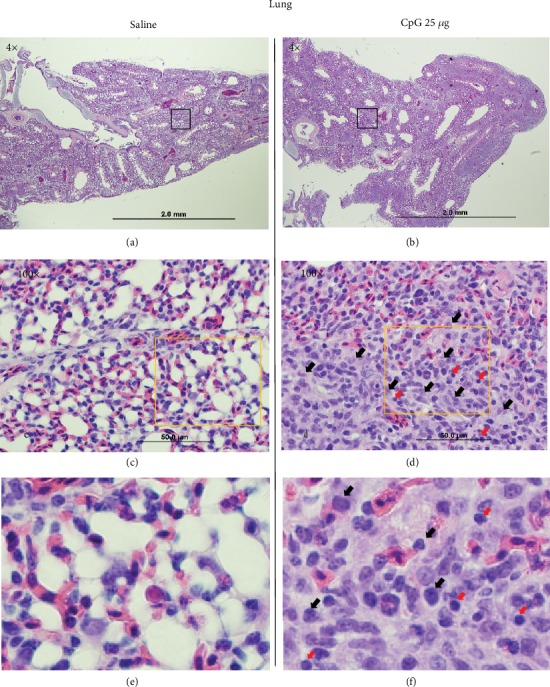
Histology of the lung. Hematoxylin and eosin staining 72 hrs post *in ovo* injections revealed higher cellular infiltration in the lungs of birds treated with 25 *μ*g CpG-ODN compared to saline control. Higher cellular infiltration was seen with CpG-ODN treatment (b) compared to saline (a) in low power (4x). High power magnification (100x) (d) and the magnified inset (f) showed that these cells mainly comprised of a mononuclear (macrophages) cell population (black arrows) and lymphocytes (red arrows).

Hematoxylin and eosin staining 72 hrs post *in ovo* injections revealed higher cellular infiltration in the lungs of birds treated with 25 *μ*g CpG-ODN (Figures 2(b), 2(d), and 2(f)) or 50 *μ*g CpG-ODN (data not shown), compared to the saline-treated group (Figures 2(a), 2(c), and 2(e)). The lungs of birds treated with CpG-ODN showed higher cellularity in the lung parenchyma (Figures 2(b), 2(d), and 2(f)), compared to saline-treated birds (Figures 2(a), 2(c), and 2(e)). Higher magnification of histology showed that these inflammatory cells were predominantly mononuclear cells with occasional heterophils (Figures 2(d) and 2(f)). There were no other inflammatory changes detected histologically suggesting no adverse effects or pathology is caused by any dose of CpG-ODN we used for this study.

### 3.3. Immune Cell Profiling of the Spleen

#### 3.3.1. Antigen-Presenting Cells in the Spleen

**Figure 3:**
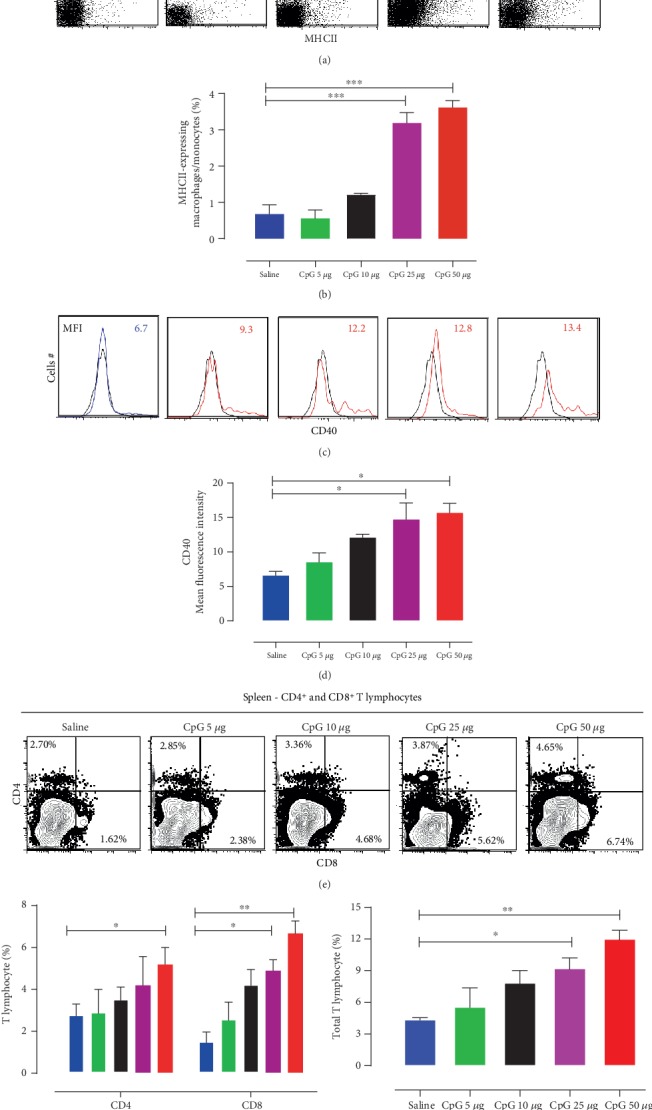
CpG-ODN induced a dose-dependent enrichment of splenic immunological niches. CpG-ODN treatment resulted in an increase in the number of infiltrating antigen-presenting cells and T lymphocytes in the spleen. Flow cytometric analysis of splenocytes at 72 hrs post *in ovo* injections (day 21 embryo) (*n* = 3‐4/group). Cells were plotted based on forward and side scatters to gate monocyte/macrophage populations. Then, using PE-labeled mouse anti-chicken monocyte/macrophage (clone: KUL01) and AF488-labeled mouse MHC II monoclonal antibodies, the double-positive population was gated to quantify the number of APCs (dot plot panels, a). Bar diagrams (b) show the means of the total percentage of MHC II^+^ APC in various groups. Histogram panels (c) indicate the level of CD40 expression on MHC II^+^ APCs (the MFI is indicated). Black histogram = isotype control, blue = saline, and red = CpG-ODN treated. Bar diagrams (d) show the means of MFI of CD40 expression on MHC II^+^ APC (vertical line and horizontal bar show the standard error of mean-SEM), *n* = 3‐4. Dunnett's test following ANOVA testing was used to test for significant differences between CpG-ODN doses and the saline control group. Asterisks indicate groups that were significantly different from the control group (*P* < 0.05). (MFI—geometric mean fluorescence intensity). (e–g) Flow cytometric analysis of the spleen T-cell populations in each group (*n* = 3‐4) at 72 hr post *in ovo* injections (day 1 hatch). CD4^+^ and CD8^+^ T-cells in the spleen (dot plot panels, e) were quantified using PE-labeled mouse anti-chicken CD4 and FITC-labeled mouse anti-chicken CD8 monoclonal antibodies. Bar diagrams show CD4^+^ and CD8^+^ T-cell number in the spleen (f), and the total number of CD4^+^ T-cells and CD8^+^ T-cells combined (g) in various groups. Two-way ANOVA following the Bonferroni posttest was done when CD4^+^ and CD8^+^ cells were compared between CpG-ODN received groups and saline control. Dunnett's test following ANOVA testing was used to test for significant differences in total T-cells between different CpG-ODN doses and the saline control group. Vertical line and horizontal bar show the standard error of mean-SEM. Asterisks indicate groups that were significantly different from the control group, ^∗^*P* < 0.05, ^∗∗^*P* < 0.01, and ^∗∗∗^*P* < 0.001.

Flow cytometry of splenocytes demonstrated the immune cell profile pattern similar to the lung samples. We observed a substantial effect of CpG-ODN treatment on MHCII-expressing APC cell population (monocyte/macrophages) in the spleen 72 hrs post *in ovo* injections. The groups that received 25 *μ*g or 50 *μ*g of CpG-ODN per bird showed a significant increase in the splenic APC population compared to the saline control group (~3 and ~5 times higher, respectively) (Figures 3(a) and 3(b)). In contrast, 5 *μ*g and 10 *μ*g of CpG-ODN treatment did not show a significant effect on APC population compared to the saline-treated control group in the lungs (Figures 3(a) and 3(b)). The MFI of CD40 expression on splenic APCs, with each CpG-ODN dose tested, are shown in histograms (Figure 3(c)) and in the graph (Figure 3(d)). The MFI increased in a dose-dependent manner and was significantly high in both 25 *μ*g and 50 *μ*g groups (Figure 3(d)).

#### 3.3.2. CD4^+^ and CD8^+^ T-Cell Subsets in the Spleen

We evaluated the effect of CpG-ODN on CD4^+^ and CD8^+^ cell populations in the chicken spleen 72 hrs post-CpG-ODN *in ovo* injections. We found a dose-dependent increase in both CD4^+^ and CD8^+^ cell populations in the spleen after CpG-ODN in ovo administration (Figures 3(e)–3(g)). A significant increase in CD4^+^ T-cells (in 50 *μ*g group) and CD8^+^ T-cells (both 25 *μ*g and 50 *μ*g groups) was observed (Figure 1(f)). However, the total number of T lymphocytes (CD4^+^ and CD8^+^ T-cells combined) (Figure 3(g)) was significantly high in the spleen of chickens that received 25 *μ*g or 50 *μ*g CpG-ODN.

#### 3.3.3. Histological Evaluation

**Figure 4:**
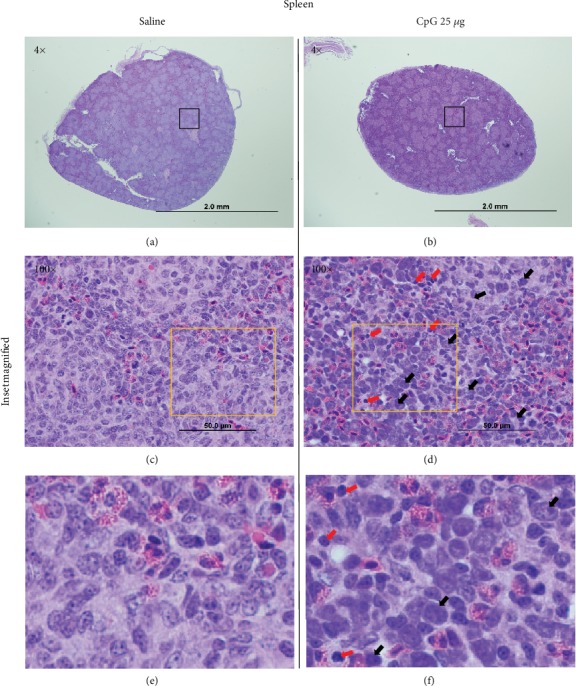
Histology of the spleen. Hematoxylin and eosin staining 72 hrs post *in ovo* injections revealed higher cellular infiltration in the spleen of birds treated with 25 *μ*g CpG-ODN compared to saline control. Higher cellular infiltration was seen with CpG-ODN treatment (b) compared to saline (a) in low power (4x). High power magnification (100x) (d) and the magnified inset (f) showed that these cells mainly comprised of a mononuclear (macrophages) cell population (black arrows) and lymphocytes (red arrows).

Hematoxylin and eosin staining 72 hrs post *in ovo* injections revealed higher cellular infiltration in the spleen of birds treated with 25 *μ*g CpG-ODN (Figures 4(b), 4(d), and 4(f)) or 50 *μ*g CpG-ODN (data not shown), compared to the saline-treated group (Figures 4(a), 4(c), and 4(e)). Higher magnification of histology showed that these inflammatory cells were predominantly mononuclear cells with occasional heterophils (Figures 4(d) and 4(f)). We did not observe any adverse effect or pathology in this study.

### 3.4. *E. coli* Challenge

**Figure 5:**
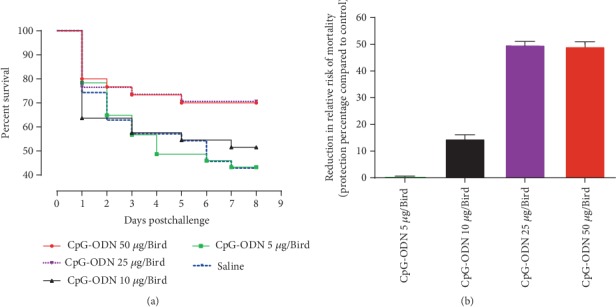
Survival pattern and reduction of the relative risk of mortality following the *E.coli* challenge. (a) Survival of broiler chickens following *E. coli* challenge. Groups of broiler chicken embryos at day 18 of incubation were injected with either 5, 10, 25, and 50 *μ*g of CpG-ODN or sterile saline by the *in ovo* route and then challenged with either 1 × 10^5^ or 1 × 10^4^ cfu of *E.coli* three days later, at the day of hatch. Survival graph was plotted based on the event of a death in birds following challenge for each day postchallenge in each group. Survival was significantly higher in the birds that received 25 *μ*g or 50 *μ*g of CpG-ODN compared to the group of birds that received saline (*P* = 0.0323 and 0.0430, respectively). (b) The reduction of the relative risk of mortality compared to the saline control group following *E. coli* challenge.

The data of groups that received either 1 × 10^4^ or 1 × 10^5^ cfu of *E. coli* were combined for clarity to present both the survival and the clinical score (CCS) analysis. In this study, survival following the *E. coli* challenge was significantly higher in groups of birds that received 25 *μ*g or 50 *μ*g of CpG-ODN compared to the group of birds that received saline (*P* = 0.0323 and 0.0430, respectively) (Figure 5(a)). Moreover, the reduction in the relative risk of mortality following the *E. coli* challenge was 48.5% and 47.5%, respectively, in these group, indicating that increasing the dose beyond 25 *μ*g did not improve survival. This study revealed that the level of protection following the *E. coli* challenge is similar in birds that received 25 and 50 *μ*g of CpG-ODN (Figure 5(b)). Birds that received 10 *μ*g of CpG-ODN tended to have higher survival than the saline control birds but were not statistically significant (*P* = 0.5957). The survival of birds that received 5 *μ*g of CpG-ODN was similar to the saline control group (Figure 5(a)).

**Figure 6:**
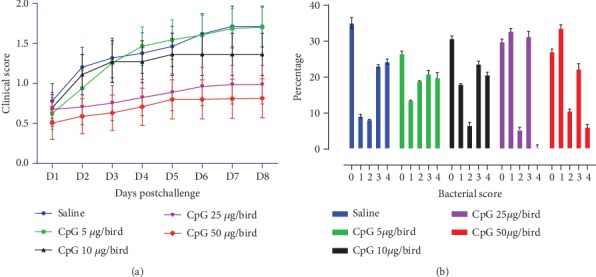
Clinical scores and bacterial growth following the *E. coli* challenge. (a) Clinical scores (CCS) of broiler chickens following *E. coli* challenge. Each data point represents the CCS of the individual group at that point (bar = mean with SEM). A significantly less CCS was recorded in 25 *μ*g (*P* = 0.0025), and 50 *μ*g (*P* = 0.0016) of CpG-ODN treated groups, compared to the saline-treated group. (b) Bar graph shows the percentage of birds in each treatment group that had each classification of bacterial growth. Bacterial swabs were taken from air sacs. Groups that received 25 *μ*g and 50 *μ*g of CpG-ODN showed significantly (*P* < 0.0001) less number of birds with higher levels (4+) of bacterial growth compared to other groups.

The CCS for each bird was calculated by summing the daily scores throughout the 7-day observation period after the *E. coli* challenge. A significantly less CCS was recorded in 25 *μ*g (*P* = 0.0025) and 50 *μ*g (*P* = 0.0016) of CpG-ODN-treated groups, compared to the saline-treated group (Figure 6(a)). The lowest CCSs were seen in birds that received 50 *μ*g of CpG-ODN while the highest CCSs were detected from the saline control group (Figure 6(a)). Moreover, birds that received CpG-ODN had lower amounts of bacteria isolated from air sac swabs compared to birds that received saline (Figure 6(b)). In comparison to other groups, birds that received 25 *μ*g or 50 *μ*g of CpG-ODN had significantly (*P* < 0.0001) less number of birds with 4+ levels of bacterial growth. Birds that died or were euthanatized either had airsacculitis or pericarditis or a combination of airsacculitis together with pericarditis or polyserositis.

## 4. Discussion

While many preventive strategies are being implemented to minimize infectious diseases in poultry, they may not be sufficient to avert all pathogenic insults. Immune-based methods that stimulate innate immunity against a broad range of pathogens may provide a promising solution to disease problems in the poultry industry. CpG-ODN, a TLR agonist, is well known as an immune protective agent against bacterial [28, 29], viral [44, 45], and protozoal [25] infections in chickens. Furthermore, several studies have shown that CpG-ODN is effective as an immunostimulant in both mature and neonatal chickens against *E. coli* infections [4, 26, 30]. We have previously shown that CpG-ODN delivered through *in ovo* route, a most desirable and economical method of drug delivery in the chicken industry [46], can be effective in protecting neonatal chickens against bacterial diseases such as those caused by *E. coli* [26, 30] and *Salmonella* Typhimurium [29]. Other studies also reported the immunoprotective effect of CpG-ODN against *Salmonella enteritidis* [31, 32] and *Campylobacter* sp. [34]. The formulation of CpG-ODN with nanoparticles further improves the immune protective effect [30, 33, 34]. A recent study using boron nitride nanospheres functionalized with mesoporous silica demonstrated enhanced the delivery of CpG ODNs into macrophages that induced higher amounts of cytokines without cytotoxicity [47]. Previous studies suggested that enhanced expression of cytokines and chemokines [34, 35] and increased cellular functions, such as the increase in heterophil degranulation and oxidative burst [48], play important roles in CpG-ODN-induced protection in chickens. We have recently reported an enhanced infiltration of inflammatory cells following an intrapulmonary delivery of CpG-ODN [4] and an enhanced enrichment of immunological niches after *in ovo* administration of CpG-ODN [40] in chickens. Despite recent progress, the manner that CpG-ODN alone confers immunoprotection against bacterial infection remains poorly understood.

The present study was undertaken to investigate CpG-ODN-mediated immune cell recruitment in the immunological niches in lymphoid (spleen) and nonlymphoid (lungs) organs in chickens and examine further if the immunological profiles correlate with protection against *E. coli* infection in chicks. In this study, we administered various doses of CpG-ODN through *in ovo* route (18-day-old embryonated eggs) and harvested the spleen and lungs at hatch to investigate CpG-ODN dose effect on the immunological profiles. One day after the hatch (four days after the CpG-ODN treatment), chicks were challenged with a virulent strain of *E. coli* to test protection from disease. Here, we examined the recruitment of macrophages/monocytes and CD4^+^ and CD8^+^ T-cell subsets in the immunological niches in the spleen and lungs. APCs, such as macrophages/monocytes, constitute essential components in the immune system, which process and present antigens, and initiate activation of effector immune cells. Maturation of APCs such as macrophages and dendritic cells involves an increased expression of costimulatory molecules such as CD80, CD86, and CD40 [49]. CD40 signaling on APC results in APC licensing (full activation) that facilitates CD8^+^ T-cell priming [43] to orchestrate protective CD8^+^ cytotoxic T-cell (CTL) immunity [50]. Therefore, we examined the number of macrophages/monocytes present in the spleen and lungs as well as the expression of CD40 on these APCs in CpG-ODN treated or saline controls.

Our results showed a CpG-ODN-dose-dependent increase in the number of macrophages/monocytes in the immunological niches in both the lungs and the spleen. Moreover, we also observed an increase in CD40 expression with different doses of CpG-ODN. CD40 signaling is important for B cell proliferation, differentiation, T-cell proliferation, monocyte and dendritic cell growth, and cytokine production [51]. These data suggest that CpG-ODN administration not only enriches immune compartments with sentinel cells such as macrophages/monocytes that play important roles fighting against pathogens [52] but also activates them for proper maturation leading to the expression of a costimulatory molecule, CD40, which is well known for its role in the orchestration of immunity against pathogens [53]. These findings seen with CpG-ODN, such as immune cell activation and maturation, were comparable to the results of many earlier studies in other species [54–57]. The significant increase we observed in MHCII-expressing APCs with 25 *μ*g and 50 *μ*g of *in ovo* CpG-ODN in both the lungs and the spleen could suggest enhanced antigen presentation capability for an effective and rapid pathogen clearance in neonatal chicks. Also, we observed a dose-dependent increase in CD4^+^ and CD8^+^ T-cell populations, which play important roles in humoral and cell-mediated immunity, in the spleen and lungs following CpG-ODN administration. These findings suggest that the immunological niches in CpG-ODN-treated chicks are well equipped with mature APCs with the ability to activate effector immune cells such as CD4^+^ and CD8^+^ T lymphocytes. The increase in the number of immune cells in the lungs and spleen can be attributed to either CpG-ODN-induced enhanced cytokine-chemokine-mediated recruitment of immune cells or increased production of immune cells or both. We recently demonstrated that *in ovo* delivery of CpG-ODN enhanced the expression of proinflammatory cytokines, interleukin- (IL-) 1*β*, IL-6, IL-18, and lipopolysaccharide-induced tumor necrosis factor (LITAF) in the lungs and spleen, suggesting that CpG-ODN promotes inflammatory responses in chickens. Accumulating evidence indicates that proinflammatory cytokines play a critical role in promoting strong antimicrobial immune responses.

Next, we investigated whether the levels of enrichment of immunological niches in the spleen and lungs by different doses of CpG-ODN have any correlation with the immunoprotection against *E. coli* infection. Therefore, we have assessed the clinical protection of neonatal chickens treated with various doses of CpG-ODN by challenging them with a virulent strain of *E. coli*. Here, we noted that the protection level of chicks that received 10 *μ*g or less CpG-ODN was not different from the chicks that received saline, consistent with our previous studies [4, 26, 28, 58]. Chicks that received 25 *μ*g and 50 *μ*g of CpG-ODN were significantly protected compared to chicks that received saline. This protection correlates with the changes we detected in immune cell compartments in chickens at the time of hatch by different doses of CpG-ODN. We observed that 10 *μ*g, but not 5 *μ*g, CpG-ODN dose increased immune cells both in the spleen and in the lungs. However, both 5 and 10 *μ*g doses were not significantly different than the saline controls in immune profiles and protection. In contrast, chicks that received 25 *μ*g and 50 *μ*g of CpG-ODN demonstrated significantly enhanced MHCII-expressing APCs, CD40 expression on these APCs and T-cell populations that correlated with their ability to resist *E. coli* infection. Overall, our data established a cause and effect relationship between CpG-ODN-induced immune enrichment and the antibacterial immunity and suggest that CpG-ODNs not only stimulate immune cells but also remarkably enriches the immunological niche, making neonatal chicks well prepared to tackle pathogenic insults.

## Data Availability

The data used to support the findings of this study are available from the corresponding author upon request.

## Abbreviations

CpG-ODN: Oligodeoxynucleotides containing CpG motifs
PAMPs: Pathogen-associated molecular patterns
PRR: Pattern recognition receptors
TLRs: Toll-like receptors
DC: Dendritic cells
DMEM: Dulbecco's modified Eagle medium
APCs: Antigen-presenting cells
CCSs: Cumulative clinical scores
CTL: CD8^+^ cytotoxic T-cell.
